# Elderly People’s Access to Emergency Departments during the COVID-19 Pandemic: Results from a Large Population-Based Study in Italy

**DOI:** 10.3390/jcm10235563

**Published:** 2021-11-26

**Authors:** Andrea Bardin, Alessandra Buja, Claudio Barbiellini Amidei, Matteo Paganini, Andrea Favaro, Mario Saia, Vincenzo Baldo

**Affiliations:** 1 Department of Cardiologic, Vascular and Thoracic Sciences, and Public Health, University of Padova, 35128 Padova, Italy; andreabardin12@gmail.com (A.B.); claudioamidei@gmail.com (C.B.A.); vincenzo.baldo@unipd.it (V.B.); 2 Department of Biomedical Sciences, University of Padova, 35131 Padova, Italy; matteo.paganini@unipd.it; 3 Emergency Department and Emergency Medical Service, San Bassiano Hospital, ULSS 7 “Pedemontana”, 36061 Bassano del Grappa, Italy; andrea.favaro@aulss7.veneto.it; 4 Clinical Governance Unit, Azienda Zero, 35131 Padova, Italy; mario.saia@azero.veneto.it

**Keywords:** emergency department, pandemic, health care services, lockdown, COVID-19, elderly population

## Abstract

Across the world, people have avoided seeking medical attention during the coronavirus pandemic, resulting in a marked reduction in emergency department (ED) visits. This retrospective cohort study examines in detail how the present pandemic affects ED use by the elderly. The regional database on ED visits in Veneto (northeastern Italy) was consulted to extract anonymous data on all ED visits during 2019 and 2020, along with details concerning patients’ characteristics (access mode, triage code, chief complaint, and outcome). A year-on-year comparison was drawn between 2019 and 2020. There was a 25.3% decrease in ED visits in 2020 compared to the previous year. The decrease ranged from −52.4% in March to −18.4% in September when comparing the same months in the two years. This decrease started in late February 2020, with the lowest numbers of visits recorded in March and April 2020 (during the “first wave” of the COVID-19 pandemic in Italy), and in the autumn (during the “second wave”). The proportion of visits to the ED by ambulance has increased sharply since March 2020, and patients arrived more frequently with severe conditions (red or yellow triage tags) that often required a hospitalization. The greatest decrease was in fact observed for non-urgent complaints. This decreased concerned a wide range of conditions, including chest pain and abdominal pain. The sharp reduction observed in the present study is unlikely to be attributed entirely to the effect of lockdown measures. Individual psychological and media-induced fear of contagion most likely played a relevant role in leading people to avoid seeking medical attention.

## 1. Introduction

Since the first confirmed case of COVID-19 was recorded in China in December 2019, the SARS-CoV-2 virus has infected hundreds of millions of people worldwide, and caused the deaths of several millions of people [1]. Italy in particular was badly affected by the pandemic during the spring and autumn of 2020, and by the end of December 2020, the country had recorded over 2.1 million cases and 77,583 deaths [2].

The first clusters of cases were detected in Italy in late February 2020 [3]. Strict restrictions on social contacts and individual mobility were adopted all over the country on March 10th, placing more than 60 million people under lockdown to combat the spread of COVID-19 and contain the increasing strain on hospital-based services, especially intensive care units (ICUs). From late May 2020 onwards, the situation improved and many restrictions were gradually eased, with freedom of movement across Italian regions and other European countries fully restored during the summer. In October, Italy and the rest of Europe were hit by a second wave of the pandemic, which induced governments to reintroduce restrictions on people’s movements and social lives. As the epidemic spread, ICUs and hospitals dedicated to COVID-19 patients experienced a surge in admissions, while access to other healthcare services decreased. Soon after the COVID-19 pandemic began early in 2020, the impact of the pandemic and the measures imposed on the provision and usage of healthcare services included a decline in appointments with General Practitioners (GPs), specialist consultations, and hospital admissions [4,5], as well as fewer visits to Emergency Departments (EDs) in badly affected countries [6,7,8] The increased number of non-COVID-related deaths at home during the first wave of the pandemic in Italy also suggests that the use of EDs decreased at that time, even among those cases where a serious illness was involved [9]. This may be partly due to public health authorities recommending that people self-isolate and stay at home, to avoid overburdening the hospitals. These recommendations may have affected the behaviour of the elderly, a demographic group known to be especially vulnerable to COVID-19 ever since the start of the pandemic [10,11]. On the other hand, the elderly are especially influenced by the fear of becoming infected with the virus [12], and this may have made them more reluctant to visit the ED even in times of need. A reduction in ED visits involving the older population was reported in the USA [13], though extensive studies specifically investigating this age group are lacking. The present study aimed to analyze in depth the ED visits made in 2019 and 2020 by people aged 65 and over in Veneto, a large region of northeastern Italy. 

## 2. Methods

### 2.1. Context

In Italy, healthcare is provided to all citizens and residents by a mixed public–private system. It is funded by the taxpayer and administered on a regional basis, under regulations issued by the national Ministry of Health. The present study focused on Veneto, which has a population of 4.9 million, of which 23.3% is 65 or older (compared to the national figure of 23.2%). This region is one of the wealthiest in Italy, with a GDP per capita of 116% that of the rest of Italy [14]. During 2019 and 2020, there were 52 active EDs in the region: 46 public and 6 private. The region’s healthcare facilities are interconnected, forming a regional hospital network that comprises: (a) 7 major “hub” hospitals, including 2 university hospitals, with highly specialized services located in the main cities; (b) 24 medium-sized “spoke” hospitals, each serving an average population of 250,000; and (c) 21 small local hospitals. Veneto was among the Italian regions that were affected the most by COVID-19, but its early public health response, including thorough case finding and contact tracing, and a substantial increase in ICU capacity, helped to prevent any breakdown of the regional healthcare system [3,15]. When the pandemic hit in February 2020, Italy was the first European country to introduce drastic containment measures: by the end of February 2020, schools were closed in many Italian regions, including Veneto; on March 10th, a lockdown was enforced across the whole country, limiting social movements to strict necessities. From the beginning of May to 14 June, containment measures were progressively relaxed. Then, new restrictions were introduced again in October at the beginning of the so called “second wave”, differentiating stringency on a regional basis, with Veneto maintaining at least some restrictions throughout all autumn 2020.

### 2.2. Materials

The present study has a retrospective cohort design. Records of all ED visits made by elderly people in 2019 and 2020 were obtained, allowing for the collection of information on patients’ date of access, arrival mode, age and gender, clinical presentation, triage code, and outcome of the ED visit. Three age brackets were considered: 65 to 74, 75 to 84, and over 84 years. Access mode was classified as either walk-in or by ambulance. Clinical presentations (chief complaints) on arrival were classified in one of the following 23 categories: abdominal pain; acute neurological syndrome; allergic reactions; burn or trauma; chest pain; coma; dermatological symptoms; dyspnea; ear, nose and throat disorders; fever; forensic/legal medicine; gynecological disorders; hypertension; nephrological-urological disorders; nontraumatic hemorrhage; odontostomatological diseases; ophthalmological symptoms; other nervous system symptoms; other symptoms; poisoning; shock; social problems; and tachycardia and palpitations. This list was derived from the regional standardized triage system, with only minor adjustments necessary to assure comparability along the two-year period we analyzed. Triage codes were tagged as follows: white tag [Wt] (not urgent); green tag [Gt] (low priority); yellow tag [Yt] (urgent, potentially life-threatening); red tag [Rt] (critical, life-threatening). The outcomes of ED visits were classified as “discharge home” (patient is eligible for outpatient care after ED assessment), “hospitalization” (patient is hospitalized after ED assessment), or “death” (patient died in the ED). Among the records of all ED visits made in Veneto in the two years considered (614,612 in 2019, and 458,813 in 2020), the patient’s age was missing in 79 cases in 2019, and 3951 cases in 2020. These records, representing less than 0.5% of the total records, were excluded from all our analyses. Finally, data on confirmed cases of COVID-19 and patients hospitalized with symptoms suggestive of the disease were retrieved from the Italian Civil Protection Department’s database for monitoring COVID-19 pandemic [16].

### 2.3. Statistical Analysis

The number of ED visits per month in 2020 was compared with the number of new daily confirmed cases of SARS-CoV-2 infection and hospitalizations of patients with symptoms suggestive of COVID-19 in Veneto, in 2020.

The numbers of ED visits in 2019 and 2020 were compared, stratifying by: gender, age bracket, month of the year, triage code, and clinical presentation. The bimonthly trend of ED visits for the 10 most frequent clinical presentations (over the whole study period) was then calculated for 2019 and 2020. The year-on-year change (comparing the same months in 2019 and 2020) in the number of ED visits per month was measured, both as a whole and as stratified by triage code. The proportion of visits by access mode, triage code, and outcome was calculated for pre-pandemic times (from January 2019 to February 2020) and pandemic times (from March 2020 to December 2020) and analyzed descriptively. All analyses were run with R statistical software version 4.1.1 (Auckland, New Zealand) and Microsoft Excel.

### 2.4. Ethical Statement

The study was conducted using data routinely collected by the healthcare services in anonymized records. The data analysis was performed on aggregate data. All data in the Local Health Authority registries are recorded with the patient’s consent and can be used as aggregate data for scientific studies without further authorization (Garante per la protezione dei dati personali, Resolution n.85 of 1 March 2012). This study complies with the Declaration of Helsinki and the Italian Decree n.196/2003 on personal data protection.

## 3. Results

**Figure 1:**
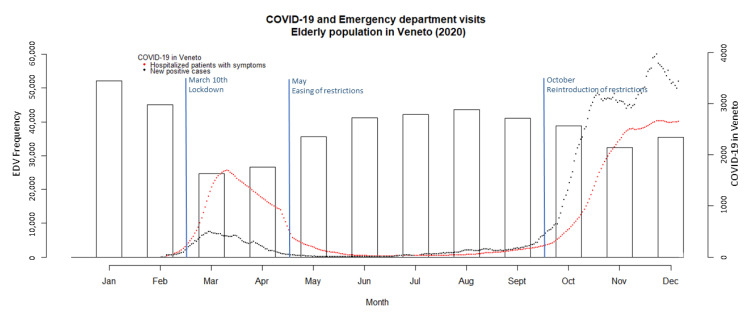
Trend of COVID-19 pandemic and emergency department visits in 2020 in Veneto (Italy). The number of ED visits is shown on the y-axis (left), and its monthly trend is represented by the vertical bars (histogram); the number of new COVID-19 cases and hospitalizations of patients with symptoms of COVID-19 is shown on the right y-axis, and their trends over time are represented by black and red dots, respectively.

Considering only patients aged 65 or over, there were 614,612 ED visits in Veneto in 2019, and 458,813 in 2020, corresponding to a 25.3% decrease. Figure 1 shows the trend in ED visits over the course of 2020, together with the trend of the COVID-19 pandemic (as captured by new daily confirmed cases of SARS-CoV-2 infection and hospitalizations of patients with COVID-19 symptoms). There was a sudden decrease in ED visits in March and April 2020, coinciding with the so-called “first wave” of the COVID-19 pandemic in Europe. This was followed by a gradual return to ordinary monthly levels of ED visits during the summer, followed by a decrease again in the autumn with the arrival of the second wave of the pandemic.

Table 1 shows a comparison between ED visits in 2019 and 2020 stratified by gender, age bracket, month of the year, and triage code. The proportion of females among patients accessing EDs decreased slightly, from 53.9% in 2019 to 52.4% in 2020. The age of patients visiting the ED did not change significantly, with 24.1% of all ED visits involving patients over 84 years old in 2019 and 24.7% in 2020.

**Figure 2:**
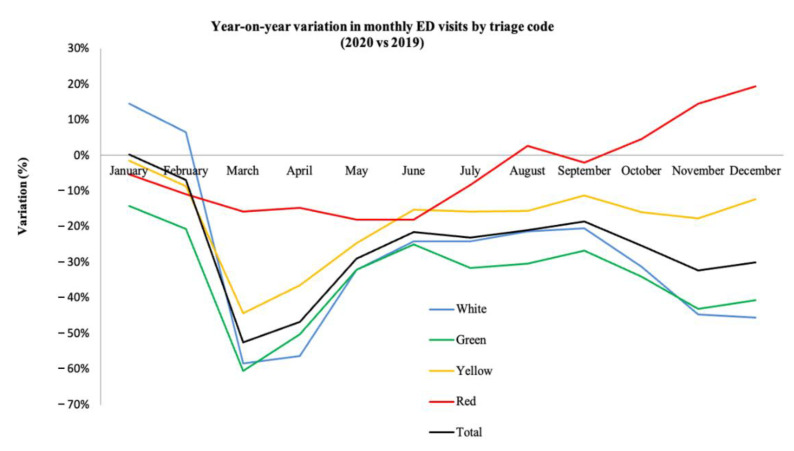
Year-on-year change (%) in the number of monthly emergency department (ED) visits 2019–2020, by triage code and overall. Orange triage codes introduced in 2020 were merged with yellow codes for consistency with the rest of the study period.

From a month-by-month comparison between the two years (Figure 2), the most significant decrease in ED visits occurred in March 2020 (down 52.4%), followed by April (down 46.3%). With the exception of January, when ED visits in 2020 were slightly higher than in 2019 (+0.4%), ED access rates were always lower in 2020. It is noteworthy that there was already a modest but significant decrease (−6.9%) in February. Comparing 2019 with 2020 by triage code, the largest reduction in access rates concerned the white and green codes, down 28.6% and 33.9%, respectively. ED visits for more severe conditions (labelled with yellow and red codes) also decreased, but to a lesser degree (down 18.2% and 4.4%, respectively).

Table 2 shows the number of ED visits by clinical presentation (chief complaint) in 2019 and 2020, and the percentage variation between the two years. With a substantial reduction in the number of ED visits recorded for all clinical presentations from 2019 to 2020, the proportion of specific clinical presentations in 2019 and 2020 remained roughly the same, with the exception of “fever” (from 1.7% to 2.6%), and dyspnea (from 9.1% to 10.9%). The largest reductions concerned “odontostomatological diseases” (−38.46%), “ophthalmological symptoms” (−38.28%), “allergic reactions” (−37.55%), “ear nose & throat disorders” (−36.85%), and chest pain (−36.14%).

**Figure 3:**
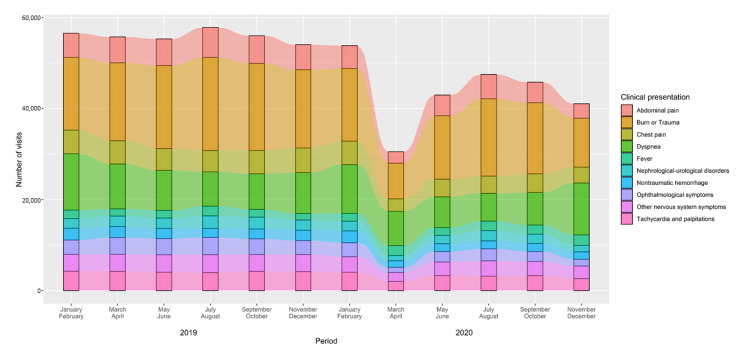
Trend in number of emergency department visits for the most common clinical presentations (chief complaints), 2019–2020 among people aged 65 or over. Not specified or missing clinical presentations were excluded from the analyses.

The trend in ED visits for the ten most common clinical presentations during the two years considered is shown in Figure 3, which breaks down the 2019–2020 period into bimesters. This figure highlights the marked decrease in the number of visits in March–April 2020—except for “fever” and “dyspnea”. ED visits for fever went from being the tenth to the fifth most common clinical presentations in these two months, descreasing back to almost ordinary levels in the summer, then increasing again to become the sixth most common in November–December 2020, during the “second wave” of the pandemic. Visits for dyspnea also increased at the end of 2020, becoming the most common complaint in November–December.

**Figure 4:**
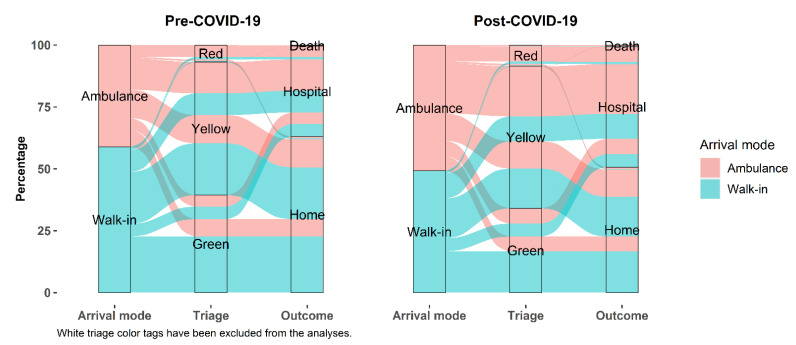
Proportion of emergency department visits by access mode, triage code, and outcome, in the pre-pandemic period (briefly referred to as “Pre-COVID-19”, from January 2019 to February 2020) and after the pandemic started (briefly referred to as “Post-COVID-19”, from March 2020 to December 2020). Visits with a white triage code were removed to better appreciate the most relevant changes between the two years for more severe medical conditions (i.e., red, yellow, and green triage codes). Any ED trajectory that included missing data on arrival or discharge mode, or any trajectory that included a white color code was excluded from the analyses (*n* = 414,539). Orange color codes introduced in 2020 were converted into yellow codes for consistency with the rest of the study period.

Figure 4 summarizes the proportion of ED visits in pre-pandemic times (from January 2019 to February 2020) and pandemic times (from March 2020 to December 2020), by arrival mode, triage code, and outcome. Considering all records (white codes included), arrival by ambulance rose from 30.0% to 38.4% after the beginning of the pandemic, and so did the proportion of yellow and red triage codes combined (from 37.2% to 41.6%). Outcomes such as death (from 0.23% to 0.41%) and hospitalization (from 25.6% to 36.0%) also increased markedly after the beginning of the pandemic, while patients discharged at home decreased from 74.1% to only 63.3% of all ED visits.

Finally, in a separate analysis on ED visits, with dyspnea alone as main clinical presentation, we found a 52.3% increase in the number of deaths among patients with a red code, when comparing 2020 with 2019 (430 and 662 deaths in 2019 and 2020, respectively). We also found a slight increase (+2.4%) in the number of ED visits that resulted in hospitalization (34,260 and 35,099 hospitalizations in 2019 and 2020, respectively) among patients accessing EDs with dyspnea as the main clinical presentation.

## 4. Discussion

### 4.1. Overview on Emergency Department Visits

This study identified a significant decrease in the total number of elderly people accessing EDs in 2020 compared with 2019, mostly attributable to the COVID-19 pandemic. Previous research showed a similar trend among the general population but, as far as we know, this is the first large-scale study focusing specifically on the older population. The slight, but significant, decrease in ED visits noted in February 2020 could be a consequence of the first public health measures put into place to combat the spread of COVID-19, after the first cases were recorded in the Veneto region starting on 21 February [3]. The other fluctuations in ED presentations seen in the present study during 2020 were strongly influenced by the trend of the epidemic in the region, higher case rates of COVID-19 coinciding with fewer elderly people going to the ED. These findings are consistent with those seen in the Italian population as a whole [17,18,19], and in the older population in the USA [12]. The decrease in ED access rates disproportionally concerned less urgent cases, classified as white and green tags at triage.

This phenomenon has several possible explanations. First, it may be a consequence of national and local public health authorities advising against going to the ED in the event of fever or respiratory symptoms in the early stages of the pandemic. The lockdowns enacted to contain waves of SARS-CoV-2 virus transmission also discouraged people from seeking medical attention. This could be especially true of most of the elderly, who are frequently not self-sufficient, and travel restrictions disrupted their social networks, making it difficult for caregivers (family, friends, and professionals) to reach their patients. Reduced access to primary care support may have been responsible for a worsening of health conditions in the elderly. Fear of contagion, dissuasion of accessing the EDs, and travel restrictions combined, most likely led to the seeking of medical attention for more severe conditions. The population’s behavior may also have changed during the first and second waves of the pandemic due to a fear of contracting the disease in hospitals, and especially in EDs, as evidence from other countries and social imitation theory seem to suggest [20,21]. Finally, as it was clear since the beginning of the pandemic that vital hospital care could easily become a critical resource during a pandemic, it is likely that special attention to avoid unnecessary hospital care, for whatever condition, was given by healthcare professionals at the primary care level (e.g., treating patients at home whenever possible).

This interpretation is supported by our analysis of clinical presentations. Dermatological, odontostomatological, and ophthalmological ED visits decreased sharply in 2020, since these conditions were rarely life-threatening. At the same time, the incidence of some acute conditions most likely decreased as a result of people’s restricted mobility, as was the case for traumatic injuries [22]. That said, there was also a substantial, albeit less pronounced, reduction in ED visits for more severe conditions, such as chest pain, and for critical conditions like coma and shock. This would indicate that the fear of contagion among the elderly, combined with the other possible reasons mentioned earlier may have discouraged people from seeking medical attention for very urgent conditions and emergencies, leading to higher non-COVID-19 mortality rates and increasing deaths at home or in nursing homes, as suggested by previous studies conducted in Italy [18,23,24,25,26,27] and in the USA [28,29].

These data may also point to a misuse of EDs in 2019 (and presumably in previous years too), an issue already raised in Italy and elsewhere, and also observed among the elderly in Veneto [30]. In Italy, the elderly account for nearly one in four of all ED visits. However, this demographic could have a greater clinical complexity, with the elderly more likely to be attributed yellow or red triage codes than younger individuals [31,32], so misuse might not be the only issue worth addressing. While elderly people have a greater need for emergency healthcare than other age groups, the quality of primary care can affect access to EDs, and ambulatory care-sensitive conditions (that could be managed with timely and effective outpatient care) account for a significant proportion of ED visits [33]. 

Visits to the ED with fever as the main clinical presentation stand out as the only exception to an otherwise homogeneous decrease in access to the ED: such visits started to increase in March 2020, then followed a trend similar to that of the local COVID-19 epidemic, with a significant increase occurring again in autumn 2020, when Italy was hit by the second wave of the pandemic. Towards the end of 2020, there was also a marked increase in visits for dyspnea, another major symptom of COVID-19.

### 4.2. Outcomes of Emergency Department Visits

A much larger proportion of elderly patients arrived at the ED by ambulance in 2020 compared with the previous year, a trend already highlighted by other studies conducted in Italy on the population as a whole [18]. However, this was not a universal trend, as in other countries, such as Japan, the use of ambulances decreased in the early stages of the pandemic [34]. This could be partly explained by COVID-19 patients in severe clinical conditions (e.g., needing oxygen through portable non-invasive ventilation devices, available in ambulances). Ambulances may be preferred over private vehicles, since they could guarantee transport with a low risk of contagion compared to private transport. Ambulances were also used to transfer patients to COVID-19-dedicated hospitals. This was partly because patients with symptoms suggestive of COVID-19 were often taken by ambulance to facilities specifically designated to treat and admit COVID-19 patients, and partly to reduce the risk of contagion in private vehicles transporting such patients. It may also reflect the decrease in the number of ED visits for less urgent health conditions in 2020, consistent with the marked reduction in the low priority (i.e., white and green) triage codes we found. This self-selection of more serious conditions may well also explain the increase in the rates of hospitalization and deaths in 2020 as outcomes of patients accessing the EDs. One way this could have happened is likely to be ED visits being postponed until a patient’s clinical conditions became critical, resulting in a higher patient death rate.

### 4.3. Strengths and Limitations

One limitation of this study concerns the lack of information on patients’ comorbidities and clinical characteristics other than the clinical presentation tags assigned to them on triage when they arrived at the ED. We also lacked information about any possible changes in the incidence of specific medical conditions during the pandemic. This prevented us from drawing solid conclusions regarding the possible changes in patients’ usage of EDs during the pandemic as a result of variations in the epidemiology of specific conditions. Further studies are needed to measure such changes, both in the use of EDs and in the epidemiology of specific diseases. We also lacked data on in-hospital outcomes, which prevented us from a detailed analysis of the severity of illness among patients that were hospitalized. Moreover, as it was not possible to distinguish COVID-19 patients that accessed the emergency department from the other patients, we could not perform any analyses on this subgroup in relation to the severity of any outcome, including mortality, reducing the depth of our analysis on outcomes. Another limitation of the study was the fact that only one pre-pandemic year was used. However, there were 597,094 ED visits in 2018 among people aged 65 or older, compared to 614,612 in 2019, a 2.9% difference with a slight upward trend. This would indicate that 2019 was quite a typical year in Veneto, in terms of ED usage. Since the reduction we found in 2020 was 25.3% compared to 2019, we consider our main results to be sufficiently reliable in terms of statistical significance.

Strengths of this study include a large population-based study, with a complete set of information on ED visits recorded by Italy’s national health service. This enabled us to stratify our analyses and shed more light on specific phenomena occurring during the COVID-19 pandemic in subgroups of the population. By representing trajectories (i.e., arrival mode, triage code, and outcome), we were also able to compare ED usage paths before and during the pandemic.

## 5. Conclusions

In conclusion, our study highlighted how the spread of SARS-CoV-2 and the associated lockdown measures has produced a noticeable decrease in the number of ED visits by the elderly in Veneto since February 2020. The decline was sharper in early spring and late autumn 2020, during the first and second waves of the pandemic in Europe, and it was more pronounced for less urgent medical conditions. There was also evidence of an increase in the number of such patients arriving at the ED by ambulance, and a decrease in the number discharged home afterwards, suggesting a change in attitude towards the ED by the elderly. That said, a reduction in the number of ED visits for more serious conditions seems to suggest that some patients with life-threatening acute diseases did not seek adequate medical treatment during the most critical phases of the pandemic. Public health experts should learn from past experiences and tailor policies to counter the pandemic efficiently without hindering the quality of healthcare received by people. We need to ensure that people—and especially the elderly, who are more vulnerable—can access full medical support in the event of clinical emergencies, and be managed in safer, non-hospital facilities if their needs are not urgent. Actions taken to achieve this may include the strengthening of outpatient primary care, expanding the use of telemedicine, and guaranteeing safe, ad hoc ED paths for elderly patients.

## Figures and Tables

**Table 1:** Emergency department visits in 2019 and 2020, by gender, age, month, and triage code.

	2019		2020		∆ 2019–2020
	*n*	%	*n*	%	%
TOTAL	614,612		458,813		−25.3%
Sex *					
Male	283,302	46.1%	218,331	47.6%	−22.9%
Female	331,308	53.9%	240,482	52.4%	−27.4%
Age					
65–74 years	217,198	35.3%	161,105	35.1%	−25.8%
75–84 years	249,306	40.6%	184,369	40.2%	−26.0%
≥85 years	148,108	24.1%	113,339	24.7%	−23.5%
Month					
January	51,988	8.5%	52,187	11.4%	0.4%
February	48,408	7.9%	45,047	9.8%	−6.9%
March	51,697	8.4%	24,633	5.4%	−52.4%
April	49,526	8.1%	26,598	5.8%	−46.3%
May	50,021	8.1%	35,643	7.8%	−28.7%
June	52,383	8.5%	41,263	9.0%	−21.2%
July	54,820	8.9%	42,272	9.2%	−22.9%
August	55,397	9.0%	43,643	9.5%	−21.2%
September	50,330	8.2%	41,054	9.0%	−18.4%
October	51,876	8.4%	38,761	8.5%	−25.3%
November	47,634	7.8%	32,331	7.1%	−32.1%
December	50,532	8.2%	35,381	7.7%	−30.0%
Triage code					
Red	24,928	4.1%	23,841	5.2%	−4.4%
Yellow	200,309	32.6%	163,946	35.7%	−18.2%
Green	149,753	24.4%	99,042	21.6%	−33.9%
White	237,447	38.6%	169,624	37.0%	−28.6%
Not indicated **	2175	0.4%	2360	0.5%	8.5%
Arrival mode ***					
Ambulance	182,436	29.8%	167,738	36.9%	−8.1%
Walk-in	430,444	70.2%	287,324	63.1%	−33.2%

* Sex was missing in two records; ** Not indicated: includes ED presentations for which a visit was not performed (e.g., administrative registration); *** Arrival mode was missing in 1732 records in 2019 and in 3733 records in 2020.

**Table 2:** Number and proportion of emergency department visits by clinical presentation (chief complaint) in 2019 and 2020, and variation (%) between 2019 and 2020.

Clinical Presentation (Chief Complaint) *	2019		2020		∆ 2019–2020
	*n*	%	*n*	%	%
Dermatological symptoms	8153	1.3%	4740	1.0%	−41.9%
Odontostomatological diseases	845	0.1%	520	0.1%	−38.5%
Ophthalmological symptoms	20,685	3.4%	12,767	2.8%	−38.3%
Allergic reactions	2748	0.5%	1716	0.4%	−37.6%
Ear, nose and throat disorders	9538	1.6%	6023	1.3%	−36.9%
Hypertension	5220	0.9%	3485	0.8%	−33.2%
Forensic/legal medicine	202	0.0%	137	0.0%	−32.2%
Other symptoms	224,629	36.7%	158,084	34.7%	−29.6%
Abdominal pain	34,914	5.7%	25,049	5.5%	−28.3%
Poisoning	723	0.1%	523	0.1%	−27.7%
Gynecological disorders	1382	0.2%	1010	0.2%	−26.9%
Tachycardia and palpitations	24,727	4.0%	18,199	4.0%	−26.4%
Burn or trauma	108,187	17.7%	81,190	17.8%	−25.0%
Shock	6006	1.0%	4576	1.0%	−23.8%
Nephrological-urological disorders	14,213	2.3%	10,833	2.4%	−23.8%
Chest pain	30,384	5.0%	23,161	5.1%	−23.8%
Other nervous system symptoms	22,684	3.7%	17,715	3.9%	−21.9%
Nontraumatic hemorrhage	13,764	2.3%	10,982	2.4%	−20.2%
Acute neurological syndrome	11,149	1.8%	9166	2.0%	−17.8%
Coma	5221	0.9%	4319	1.0%	−17.3%
Dyspnea	55,430	9.1%	49,700	10.9%	−10.3%
Social problems	222	0.0%	205	0.0%	−7.7%
Fever	10,462	1.7%	12,029	2.6%	15.0%
TOTAL	611,488	100%	456,129	100%	−25.4%

* Clinical presentations in declining order of percentage decrease from 2019 to 2020.

## Data Availability

The dataset generated as part of the present study is not publicly available, but available from the corresponding author (alessandra.buja@unipd.it) on reasonable request.
